# Supplementary material for: Validity and reliability evidence of a point of care assessment of salivary cortisol and α-amylase: a pre-registered study
Source: PeerJ. 2020 Jan 8;8:e8366. doi: 10.7717/peerj.8366 (PMC6954686; doi:10.7717/peerj.8366)
Supplement: Supplemental Information 1 [file peerj-08-8366-s001.docx]

Table S1. *Overview of priors employed in Bayesian correlation analysis.*

| Hypothesis | Distributional Form | Type of Prior | Source of Background Information | Hyperparameters |
| --- | --- | --- | --- | --- |
| 1a | Uniform | Weakly Informative | Coad et al. (2015); Fisher et al. (2015) | ~U (.10, .90) |
| 1b | Uniform | Weakly Informative | Coad et al. (2015); Fisher et al. (2015) | ~U (.10, .90) |
| 1c | Uniform | Weakly Informative | Coad et al. (2015); Fisher et al. (2015) | ~U (.10, .90) |
| 2a | Uniform | Weakly Informative | Coad et al. (2015); Fisher et al. (2015) | ~U (.10, .90) |
| 2b | Uniform | Weakly Informative | Coad et al. (2015); Fisher et al. (2015) | ~U (.10, .90) |
| 2c | Uniform | Weakly Informative | Coad et al. (2015); Fisher et al. (2015) | ~U (.10, .90) |
| 3 | Normal | Highly Informative | Coad et al. (2015); Fisher et al. (2015) | ~N (.85, .005) |
| 4 | Normal | Highly Informative | Coad et al. (2015); Fisher et al. (2015) | ~N (.85, .005) |
