# Supplementary material for: Validity and reliability evidence of a point of care assessment of salivary cortisol and α-amylase: a pre-registered study
Source: PeerJ. 2020 Jan 8;8:e8366. doi: 10.7717/peerj.8366 (PMC6954686; doi:10.7717/peerj.8366)
Supplement: Supplemental Information 2 [file peerj-08-8366-s002.docx]

**Registered Protocol for Data Collection and Analysis**

**Footnote for Manuscript (Methods)**

As noted below, our original (pre-registered) plan was to recruit university students enrolled in an undergraduate unit on sport and exercise psychology across testings at 8:30 am and 12:30 pm. We executed this recruitment strategy as planned (15 participants @ 8:30am; 14 participants @ 12:30pm) in a first run of this study. However, due to instability issues with the buffer dilution provided to us by the supplier (iPro), we encountered several issues with the test results whereby the device produced numerous inadmissible values (i.e., NAN). This issue with the product supplied to us was identified and rectified so we had executed a second run of this study with a new sample of participants. As such, we recruited individuals from the public because the opportunity to replicate the planned study design was no longer available.

**Preface to Version 2**

After detailed discussions with a collaborating laboratory, we will be unable collect and analyse blood albumin as part of the experimental procedures outlined here because we would need to sample blood at 30 min intervals for 5 hours post exercise to obtain a reliable assessment. As a secondary focus of the original protocol, we decided to remove this aspect of the methodological protocol. We also removed the formalised exercise screening tool, as this process will be confirmed verbally rather than in written format. All other procedures remain the same as the protocol submitted on 2017-08-21 @ 11:35AM (see file name, iPro study_methods registration-FINAL). These changes have been implemented and registered prior to the first data collection point.

**Background**

When you exercise, the physiological stress placed on the body by the activity can be measured using various markers in saliva and blood. Salivary cortisol and salivary a-amylase (pronounced alpha-amylase) are two such markers that health professionals can use to assess the psychological and physiological stress experienced. Typically, these markers are assessed via a sample of saliva, which are taken back to a laboratory and analysed using expensive and bulky equipment. However, small portable point-of-care devices are constantly being developed to make this process easier to conduct in the field. Our aim in this study is to test the reliability and validity of a device that might allow us to measure cortisol and a-amylase in the field more quickly than the laboratory methods.

**Research Question**

1. To what extent does a point of care assessment of salivary cortisol and salivary a-amylase correlate with a laboratory approach (i.e., enzyme-linked immunosorbent assay; ELISA) at pre-exercise (resting baseline), immediately post-exercise, and 30 min post-exercise?
2. Does a point of care assessment of salivary cortisol and salivary a-amylase provide a reliable and valid assessment of salivary cortisol and salivary a-amylase in the morning and early afternoon?

**Hypotheses**

Each of the following hypotheses are expected for assessments taken in the morning and early afternoon.

1. For salivary cortisol, point of care assessment will correlate positively and moderately (*r* ~ .50) with a laboratory-based assessment (a) pre-exercise (resting baseline), (b) immediately post-exercise, and (c) 30 min post-exercise.
2. For salivary a-amylase, point of care assessment will correlate positively and moderately (*r* ~ .50) with a laboratory-based assessment (a) pre-exercise (resting baseline), (b) immediately post-exercise, and (c) 30 min post-exercise.
3. Duplicate samples collected concurrently of the point of care assessment of salivary cortisol will correlate positively and strongly (*r* ~ .80).
4. Duplicate samples collected concurrently of the point of care assessment of salivary a-amylase will correlate positively and strongly (*r* ~ .80).

**Power Analysis**

This study was powered for the primary purpose of examining the association between assessment methods for physiological markers of stress. To do so, we conducted a series of Monte Carlo simulations (Muthén & Muthén, 2002) in M*plus* 8 (Muthén & Muthén, 2015). The expected effect size was informed by past work. With a sample of 25 recreational athletes, Coad, McLellan, Whitehouse, and Gray’s (2015) reported a strong correlation (*r* = .93, *p* <.001) between a point of care device (iPro; Soma Bioscience, Oxfordshire, UK) and a bio-sensory immunoassay (enzyme-linked immunosorbent assay; ELISA) for the assessment of salivary immunoglobulin (s-IgA). In a similar study, Fisher, McLellan, and Sinclair (2015) examined 15 healthy, active university students at rest and found a moderate correlation (*r* = .53, *p* = .042) between iPro and ELISA for the assessment of salivary cortisol (sCort). Both Coad et al. and Fisher et al. observed a high correlation between duplicate samples of the point of care assessment of s-IgA (*r* = .89, *p* < .001) and sCort (*r* = .89, *p* < .001) collected concurrently.

As effect sizes in published work are often overestimates of a true effect (Ioannidis, 2005), we took a conservative approach to the power simulations and worked with the lowest of the published correlation effects between assessment approaches (i.e., *r* = .50). For all simulations, both variables were standardised with a mean of 0 and variance of 1. Simulations with a robust maximum likelihood estimator (MLR) indicated that 39 participants would provide sufficient power detect a correlation of .50 between the two assessment methods (i.e., 92% power with 91% of the 95% credibility intervals of replications included the population value of .50; Muthén & Muthén, 2002). We replicated these simulations with a Bayesian estimator using a fixed number of 10,000 iterations across each of the two Markov Chain Monte Carlo (MCMC) chains. First, as a comparison with the MLR results detailed above, we started with a sample size of 41 and employed default, non-informative priors for the correlation effect. This model provided 92% power with .95 for the 95% coverage of the population to detect the expected effect of .50. With a non-informative prior, 38 participants would provide sufficient power (91% with 96% of the 95% credibility intervals of replications included the population value of .50). Second, the incorporation of a uniform prior directly within the Monte Carlo simulations where values between .10 and .90 are equally likely (and those outside this range are prohibited) indicated that 10 participants would provide 100% power and where 100% of the 95% credibility intervals of replications included the population value of .50. We preferred a uniform prior rather than an informative prior – where the mean represents the most likely expected value (e.g., μ = .50) and the variance reflects the degree of uncertainty around this estimate (e.g., σ^2^ = .04) – because of the availability of only two published studies^[[1]](#footnote-1)^. Thus, the lower and upper bounds of this uniform distribution imply that small, medium and large effects are equally likely. Based on these simulations, we decided to recruit into this study at least 15 participants to complete the study protocol in each of the testing sessions (N = 30). The M*plus* output for these Monte Carlo simulations is provided as supplementary material on the Open Science Framework (<https://osf.io/ptcn7/>).

**Planned Sample**

Participants will be university students enrolled in an undergraduate unit on sport and exercise psychology aged 18 years or older. Individuals will be excluded from taking part in the study if they report an illness or injury that prevents them from completing a sub-maximal test of aerobic work capacity.

**Materials and Procedures**

All study procedures will be completed in one testing session. Upon arriving in the laboratory and providing informed consent, participants will self-report demographic information (e.g., gender and age), lifetime adversities (adapted from Seery, Holman, & Silver, 2010), acute general (Cohen, Kamarck, & Mermelstein, 1983) and academic stressors (informed by the findings of Hurst, Baranik, & Daniel, 2013), and mental toughness level (Gucciardi et al., 2015) and stability (adapted from Dykman, 1998). The full questionnaire package is provided as an Appendix to this registration document. After completing the survey package, participants will complete a sub-maximal test of aerobic work capacity (PWC_75%HRmax_; Miyashita et al., 1985; Gore et al., 1999). Briefly, this test involves completing three workloads of 3 – 6 min at approximately 55, 65 and 75% of age predicted maximum heart rate (220 bpm – age in years) on a cycle ergometer (Monark, Monark Exercise AB, Varnsbro, Sweden). Heart rate and ratings of perceived exertion (Borg, 1982) will be collected each minute during the test. Participants will be required to avoid high-intensity exercise and alcohol for 24 h, avoid brushing their teeth or eating food for 2 h pre-exercise and will be asked to rinse their mouth with water 10 min prior to saliva collection. Owing to logistical issues beyond our control (i.e., student timetabling is managed centrally at our university), we will conduct two testing sessions at approximately 0830 and 1230.

Samples of saliva will be taken pre-warm-up, immediately post-exercise and 30 min post-exercise. A total of nine unstimulated whole saliva samples will be provided by each participant (3 x pre-warm-up, 3 x immediately post-exercise, and 3 x 30 min post-exercise). On each occasion duplicate oral swabs (iPro oral fluid collector; Soma Bioscience, Oxfordshire, UK) will be placed in the mouth concurrently for assessment using a cortisol/a-amylase lateral flow device (LFD) before being analysed using a LFD reader (iPro Cube, Soma Bioscience, Oxfordshire, UK) and process described by Fischer et al. (2015). Second, the third sample will be collected subsequently by the passive drool method directly into a 2 mL vial (Salimetrics LLC, Pennsylvania, USA). This sample will then be frozen at -20$℃$ until analysis using ELISA immunoassay.

**Analysis Plan**

A series of bivariate correlations will be performed to assess the study hypotheses detailed above. Analyses will be conducted in M*plus* 8 (Muthén & Muthén, 2015) using a Bayesian estimator and uniform prior [.10, .90] on the correlation between the measurement of salivary cortisol and a-amylase using the iPro device and ELISA method. The reliability of the point of care assessment of a-amylase and sCort collected concurrently will be assessed using an informative normal prior (μ = .85, σ^2^ = .005). These analyses and their write-up will be informed by the WAMBS checklist outlined by Depaoli and van de Schoot (2017).

Model convergence will be assessed using statistical (i.e., potential scale reduction factor; PSR < 1.05) and visual criteria (i.e., inspection of trace plots for stability in mean and variance of each chain). Posterior predictive checking is used to assess model fit in Bayesian estimation, where the posterior distribution is compared with the observed data to examine the degree to which the replicated data matches the observed data (Muthén & Asparouhov, 2012). The posterior predictive *p*-value (PPP) and associated 95% credibility interval (CI) is produced in M*plus*; values close to .50 reflect an excellent fitting model, though typically values greater than .05 are considered acceptable (Muthén & Asparouhov, 2012). Parameter estimates will be considered substantively meaningful when the 95% CI excluded zero. Missing data will be handled with the Gibbs sampler that treats the missing observations as unknown values to be estimated and the algorithm used will correctly estimate the model under the missing at random (MAR) assumption (Asparouhov & Muthén, 2010).

**References**

Asparouhov, T., & Muthén, B. (2010). *Bayesian analysis using Mplus: Technical implementation*. Retrieved from <http://www.statmodel.com/download/Bayes3.pdf>

Borgn G. (1982). Psychophysical bases of perceived exertion. *Medicine and Science in Sports and Exercise, 14,* 377-381.

Coad, S., McLellan, C., Whitehouse, T., & Gray, B. (2015). Validity and reliability of a novel salivary immunoassay for individual profiling in applied sport science. *Research in Sports Medicine, 23,* 140-150. doi: 10.1080/15438627.2015.1005300

Cohen, S., Kamarck, T., & Mermelstein, R. (1983). A global measure of perceived stress. *Journal of Health and Social Behaviour, 24,* 385-396.

Depaoli, S., & van de Schoot, R. (2017). Improving transparency and replication in Bayesian statistics: The WAMBS-checklist. *Psychological Methods, 22,* 240-261. doi: 10.1037/met0000065

Dykman, B. J. (1998). Integrating cognitive and motivational factors in depression: Initial tests of a goal-orientation approach. *Journal of Personality and Social Psychology, 74,* 139 – 158.

Fisher, R. N., McLellan, C. P., & Sinclair, W. H. (2015). The validity and reliability for a salivary cortisol point of care test. *Journal of Athletic Enhancement, 4*: 4. doi: 10.4172/2324-9080.1000204

Gore, C.J., Booth, M.L., Bauman, A., & Neville, O. (1999). Utility of pwc75% as an estimate of aerobic power in epidemiological and population-based studies. *Medicine and Science in Sports and Exercise, 31,* 348-351.

Gucciardi, D.F., Hanton, S., Gordon, S., Mallett, C. J., & Temby, P. (2015). The concept of mental toughness: Tests of dimensionality, nomological network and traitness. *Journal of Personality, 83,* 26-44*.* doi: 10.1111/jopy.12079

Hurst, C. S., Baranik, L.E., & Daniel, F. (2013). College student stressors: A review of the qualitative research. *Stress and Health, 29*, 275-285. doi: 10.1002/smi.2465

Ioannidis, J. P. A. (2005). Why most published research findings are false. *PLoS Medicine, 2,* e124. doi:10.1371/journal.pmed.0020124.

Miyashita, M., Mutoh, Y., Yoshioka, N., & Sadamoto, T. (1985). PWC_75%HRmax_: a measure of aerobic work capacity. *Sports Medicine, 2,* 159-164.

Muthén, B. O., & Asparouhov, T. (2012). Bayesian structural equation modelling: A more flexible representation of substantive theory. *Psychological Methods, 17,* 313-335.

Muthén, L. K, & Muthén, B. (1998-2015). *Mplus users guide* (7^th^ ed.)*.* Los Angeles CA: Muthén & Muthén.

Muthén, L. K., & Muthén, B. O. (2002). How to use a Monte Carlo study to decide on sample size and determine power. *Structural Equation Modelling*, *9,* 599-620. doi: 10.1207/S15328007SEM0904_8

Seery, M. D., Holman, E. A., & Silver, R. C. (2010). Whatever does not kill us: Cumulative lifetime adversity, vulnerability, and resilience. *Journal of Personality and Social Psychology, 99,* 1025-1041. doi:10.1037/a0021344

**Lifetime Adversity Scale**

We’d like to ask you about some events that may have happened to you during your lifetime. Please indicate if you have ever in your life experienced any of the following events by circling “no” or “yes”? For those events you have experienced, please indicate how many times you have experienced the event? If you cannot recall the specific number of times an event has occurred, please provide an estimation.

|  | *Have you experience this event?* | |  | *Number of times* |
| --- | --- | --- | --- | --- |
| Direct combat | No | Yes |  |  |
| Life threatening accident | No | Yes |  |  |
| Fire, flood or other natural disaster | No | Yes |  |  |
| Witness someone badly injured or killed | No | Yes |  |  |
| Rape | No | Yes |  |  |
| Sexual molestation | No | Yes |  |  |
| Serious physical attack or assault | No | Yes |  |  |
| Threatened/harassed without a weapon | No | Yes |  |  |
| Threatened with a weapon/held captive/kidnapped | No | Yes |  |  |
| Tortured or victim of terrorists | No | Yes |  |  |
| Domestic violence | No | Yes |  |  |
| Witness domestic violence | No | Yes |  |  |
| Finding dead body | No | Yes |  |  |
| Witness someone suicide or attempt suicide | No | Yes |  |  |
| Child abuse – physical | No | Yes |  |  |
| Child abuse – emotional | No | Yes |  |  |
| Any other stressful event, please specify: | No | Yes |  |  |
| 1. |  |  |  |  |
| 2. |  |  |  |  |
| 3. |  |  |  |  |
| 4. |  |  |  |  |
| 5. |  |  |  |  |
| Did you ever suffer a great shock because one of these events happened to someone close to you? | No | Yes |  |  |

**Perceived Stress Scale**

The questions in this scale ask you about your feelings and thoughts during the last week. For each statement, you are asked to indicate by circling how often you felt or thought a certain way.

| 0 | 1 | 2 | 3 | 4 |
| --- | --- | --- | --- | --- |
| *Never* | *Almost Never* | *Sometimes* | *Fairly Often* | *Very Often* |

|  | *In the last week, how often have you…* |  |  |  |  |  |
| --- | --- | --- | --- | --- | --- | --- |
| 1 | been upset because of something that happened unexpectedly? | 0 | 1 | 2 | 3 | 4 |
| 2 | felt you were unable to control the important things in your life? | 0 | 1 | 2 | 3 | 4 |
| 3 | felt nervous and ‘stressed’? | 0 | 1 | 2 | 3 | 4 |
| 4 | felt confident about your ability to handle your personal problems? | 0 | 1 | 2 | 3 | 4 |
| 5 | felt that things were going your way? | 0 | 1 | 2 | 3 | 4 |
| 6 | found that you could not cope with all the things that you had to do? | 0 | 1 | 2 | 3 | 4 |
| 7 | been able to control irritations in your life? | 0 | 1 | 2 | 3 | 4 |
| 8 | felt you were on top of things? | 0 | 1 | 2 | 3 | 4 |
| 9 | been angered because of things that were out of your control? | 0 | 1 | 2 | 3 | 4 |
| 10 | felt difficulties were piling up so high that you could not overcome them? | 0 | 1 | 2 | 3 | 4 |

**Academic Stressors**

This list includes a range of stressors that have been found to be highly applicable for university students. Thinking about your experiences over the past week, please indicate (i) how often you experienced each of these stressors and (ii) the degree to which you interpreted each as being a **challenge** (i.e., a stressor that you can potentially contribute to your personal development) or **hindrance** (i.e., interferes with your academic goals and likelihood of success) for your performance – *remember* *there are no right or wrong answers so be as honest as possible*.

| *How often did this stressor occur in the past week?* | | | | | |  | *How did you interpret this stressor?* | | | | |
| --- | --- | --- | --- | --- | --- | --- | --- | --- | --- | --- | --- |
| 0 | 1 | 2 | 3 | 4 | 5 |  | 1 | 2 | 3 | 4 | 5 |
| *No days* | *On 1 day* | *On 2 days* | *On 3 days* | *On 4 days* | *Everyday* |  | *100% hindrance*  *(interferes with academic goals)* | *75% hindrance* | *50% hindrance /50% challenge* | *75% challenge* | *100% challenge (potential to benefit personal development)* |

|  |  | ***How often did this stressor occur in the past week?*** | | | | | |  | ***How did you interpret this stressor?*** | | | | |
| --- | --- | --- | --- | --- | --- | --- | --- | --- | --- | --- | --- | --- | --- |
| 11 | Issues with your family | 0 | 1 | 2 | 3 | 4 | 5 |  | 1 | 2 | 3 | 4 | 5 |
| 12 | Issues with your partner (e.g., boy/girlfriend) | 0 | 1 | 2 | 3 | 4 | 5 |  | 1 | 2 | 3 | 4 | 5 |
| 13 | Issues with your friends | 0 | 1 | 2 | 3 | 4 | 5 |  | 1 | 2 | 3 | 4 | 5 |
| 14 | Adminstritative issues at university | 0 | 1 | 2 | 3 | 4 | 5 |  | 1 | 2 | 3 | 4 | 5 |
| 15 | Lack of time | 0 | 1 | 2 | 3 | 4 | 5 |  | 1 | 2 | 3 | 4 | 5 |
| 16 | Financial worries | 0 | 1 | 2 | 3 | 4 | 5 |  | 1 | 2 | 3 | 4 | 5 |
| 17 | Inadequate academic support from teaching staff | 0 | 1 | 2 | 3 | 4 | 5 |  | 1 | 2 | 3 | 4 | 5 |
| 18 | Lack of necessary skills | 0 | 1 | 2 | 3 | 4 | 5 |  | 1 | 2 | 3 | 4 | 5 |
| 19 | Self-expectations | 0 | 1 | 2 | 3 | 4 | 5 |  | 1 | 2 | 3 | 4 | 5 |
| 20 | Expectations of others | 0 | 1 | 2 | 3 | 4 | 5 |  | 1 | 2 | 3 | 4 | 5 |
| 21 | Academic or coursework demands | 0 | 1 | 2 | 3 | 4 | 5 |  | 1 | 2 | 3 | 4 | 5 |
| 22 | Assessable work including assignments and examinations | 0 | 1 | 2 | 3 | 4 | 5 |  | 1 | 2 | 3 | 4 | 5 |
| 23 | A disruptive or hostile learning environment | 0 | 1 | 2 | 3 | 4 | 5 |  | 1 | 2 | 3 | 4 | 5 |
| 24 | Being in an unfamiliar learning environment | 0 | 1 | 2 | 3 | 4 | 5 |  | 1 | 2 | 3 | 4 | 5 |
| 25 | Future career aspirations | 0 | 1 | 2 | 3 | 4 | 5 |  | 1 | 2 | 3 | 4 | 5 |
| 26 | Extracurricular activities (e.g., sport, paid work) | 0 | 1 | 2 | 3 | 4 | 5 |  | 1 | 2 | 3 | 4 | 5 |
| 27 | Health concerns | 0 | 1 | 2 | 3 | 4 | 5 |  | 1 | 2 | 3 | 4 | 5 |
| 28 | Diversity-related concerns (e.g., your religious views, sexual orientation, race) | 0 | 1 | 2 | 3 | 4 | 5 |  | 1 | 2 | 3 | 4 | 5 |

**Mental Toughness (level)**

Using the scale below, please indicate how true each of the following statements is an indication of how you typically think, feel, and behave – *remember* *there are no right or wrong answers so be as honest as possible*. These questions relate to you as a person and therefore in general, rather than any specific context of your life.

| 1 | 2 | 3 | 4 | 5 | 6 | 7 |
| --- | --- | --- | --- | --- | --- | --- |
| *False, 100% of the time* | *False, 85% of the time* | *False, 60% of the time* | *50/50* | *True, 60% of the time* | *True, 85% of the time* | *True, 100% of the time* |

| I believe in my ability to achieve my goals | 1 | 2 | 3 | 4 | 5 | 6 | 7 |
| --- | --- | --- | --- | --- | --- | --- | --- |
| I am able to regulate my focus when performing tasks | 1 | 2 | 3 | 4 | 5 | 6 | 7 |
| I bounce back from adversity | 1 | 2 | 3 | 4 | 5 | 6 | 7 |
| I strive for continued success | 1 | 2 | 3 | 4 | 5 | 6 | 7 |
| I can find a positive side in most situations | 1 | 2 | 3 | 4 | 5 | 6 | 7 |
| I am able to use my emotions to perform the way I want to | 1 | 2 | 3 | 4 | 5 | 6 | 7 |
| I maintain high levels of performance when challenged | 1 | 2 | 3 | 4 | 5 | 6 | 7 |
| I effectively execute my knowledge of what is required to achieve my goals | 1 | 2 | 3 | 4 | 5 | 6 | 7 |

**Mental Toughness (stability)**

Please read these statements carefully and indicate your level of agreement with each one by circling the appropriate number.

| 1 | 2 | 3 | 4 | 5 | 6 | 7 |
| --- | --- | --- | --- | --- | --- | --- |
| *Strongly Disagree* | *Disagree* | *Slightly Disagree* | *Neutral* | *Slightly Agree* | *Agree* | *Strongly Agree* |

| My level of mental toughness is something that stays pretty much the same from week to week | 1 | 2 | 3 | 4 | 5 | 6 | 7 |
| --- | --- | --- | --- | --- | --- | --- | --- |
| I notice that how I feel about my level of mental toughness changes from week by week | 1 | 2 | 3 | 4 | 5 | 6 | 7 |
| I believe I am mentally tough one week, but not so much in other weeks | 1 | 2 | 3 | 4 | 5 | 6 | 7 |
| My level of mental toughness remains the same, regardless of the demands or pressures I am confronted with | 1 | 2 | 3 | 4 | 5 | 6 | 7 |

1. We conducted a power analysis for a normal informative prior that resembled properties of the uniform prior distribution (μ = .50, σ^2^ = .04) that implies moderate effects are more likely than small or large effects; this analysis indicated that 10 participants would provide 97% power with 100% of the 95% credibility intervals of replications included the population value of .50. [↑](#footnote-ref-1)
